# Assessment of Epidermal Growth Factor Receptor (EGFR) expression in human meningioma

**DOI:** 10.1186/1748-717X-5-46

**Published:** 2010-05-30

**Authors:** A Gabriella Wernicke, Adam P Dicker, Michal Whiton, Jana Ivanidze, Terry Hyslop, Elizabeth H Hammond, Arie Perry, David W Andrews, Lawrence Kenyon

**Affiliations:** 1 Department of Stich Radiation Oncology, Weill Cornell School of Medicine at Cornell University, 525 East 68th Street, New York, New York 10065, USA; 2 Department of Radiation Oncology, Kimmel Cancer Center, Jefferson Medical College of Thomas Jefferson University, 111 South 11th Street, Philadelphia, PA 19107, USA; 3 Department of Clinical Neuroimmunology, University of Munich, Marchioninistrasse 15, Munich 81377, Germany; 4 Department of Biostatistics, Jefferson Medical College of Thomas Jefferson University, 111 South 11th Street, Philadelphia, PA 19107, USA; 5 Department of Pathology, LDS Hospital, University of Utah School of Medicine, 8th Ave. & C Street, Salt Lake City, UT 84183, USA; 6 Department of Pathology and Immunology, Washington University School of Medicine, 660 S. Euclid Avenue, Saint Louis, MO 63110, USA; 7 Department of Neurosurgery, Jefferson Medical College of Thomas Jefferson University, 111 South 11th Street, Philadelphia, PA 19107, USA; 8 Department of Pathology, Jefferson Medical College of Thomas Jefferson University, 111 South 11th Street, Philadelphia, PA 19107, USA

## Abstract

**Purpose:**

This study explores whether meningioma expresses epidermal growth factor receptor (EGFR) and determines if there is a correlation between the WHO grade of this tumor and the degree of EGFR expression.

**Methods:**

Following institutional review board approval, 113 meningioma specimens from 89 patients were chosen. Of these, 85 were used for final analysis. After a blinded review, immunohistochemical stains for EGFR were performed. Staining intensity (SI) was scored on a scale 0-3 (from no staining to strong staining). Staining percentage of immunoreactive cells (SP) was scored 1-5 (from the least to the maximum percent of the specimen staining). Immunohistochemical score (IHS) was calculated as the product of SI and SP.

**Results:**

Eighty-five samples of meningioma were classified in accordance with World Health Organization (WHO) criteria: benign 57/85 (67%), atypical 23/85 (27%), and malignant 5/85 (6%). The majority of samples demonstrated a moderate SI for EGFR. IHS for EGFR demonstrated a significant association between SI and histopathologic subtype. Also, there was a correlation between the SP and histopathologic subtype (p = 0.029). A significant association was determined when the benign and the atypical samples were compared to the malignant with respect to the SP (p = 0.009). While there was a range of the IHS for the benign and the atypical histologic subtypes, malignant tumors exhibited the lowest score and were statistically different from the benign and the atypical specimens (p < 0.001).

**Conclusions:**

To our knowledge, this represents the largest series of meningioma samples analyzed for EGFR expression reported in the literature. EGFR expression is greatest in benign meningiomas and may serve a potential target for therapeutic intervention with selective EGFR inhibitors.

## Introduction

Meningiomas represent the second most common primary central nervous system tumors, with an annual incidence in the U.S. of approximately 2.5 per 100,000 people [1]. Primary therapy for meningioma is surgical intervention, with the likelihood of recurrence inversely related to the extent of resection [2]. Unfortunately, complete resection is not always possible because of the location of these tumors near critical anatomical structures. The overall recurrence rate of meningiomas has been reported to be approximately 20%, with higher rates (30-40%) reported in patients who undergo less than complete resection (partial resection or biopsy) [3,4]. In addition, recurrence rates are higher for the more aggressive histologic variants, with 5-year recurrence rates of 38% for atypical meningiomas and 78% for malignant meningiomas [2,5].

The high recurrence rate in partially resected meningiomas has led to the use of additional therapy designed to improve tumor control. Radiotherapy is frequently administered after partial resection and has been shown to decrease or delay recurrence. The control of recurrent tumors continues to be a clinical challenge [6-8]. Currently, there are no pharmaceutical agents that are routinely used for adjuvant therapy. There is a considerable interest in evaluating new molecular markers that may also serve as potential therapeutic targets. Epidermal growth factor (EGF) is a polypeptide hormone that acts through activation of its cognate receptor (EGFR) and stimulates proliferation of a wide variety of cells in vitro and in vivo. The EGFR gene encodes a 170-kD membrane spanning glycoprotein composed of an extracellular ligand binding domain, a transmembrane region, and a cytoplasmic protein tyrosine kinase domain [9]. The EGFR is thought to play an important role in the regulation of cell division and tumor growth. In many cancers, excessive EGFR overexpression has been shown to stimulate angiogenesis, cell survival, and metastatic proliferation.

A wide variety of normal and neoplastic tissues express EGFR, and its overexpression has been detected in a number of human tumors including breast [10], lung [11], head and neck [12], glioblastoma multiforme [13,14], and colorectal carcinomas [11,15], to name a few. Recently, an interest emerged in assessing expression of EGFR in CNS malignancies such as meningiomas, gliomas, etc [16,17]. In 1987, Weisman, et al. [18], characterized expression of EGFR in meningiomas and suggested that EGFR is involved in the proliferation and/or differentiation of meningothelial cells. The present study represents the largest series evaluating EGFR expression in meningiomas in the literature to date. The primary objectives of the study are to determine if EGFR is expressed in meningioma and whether there is a correlation between the WHO tumor grade of this tumor and the degree of EGFR expression.

## Materials and methods

### Case Selection

Following institutional review board approval, a computerized search of the surgical pathology database of Thomas Jefferson University Hospital (Philadelphia, PA) and Washington University Hospital (St. Louis, MO) was performed. A total of 113 meningioma specimens from 89 patients were identified between 1995 and 2001. Of these, 85 were used from 85 patients for the final analysis and chosen for further study based on adequacy of tissue, tissue preservation, and unequivocal diagnostic features. After review of the original hematoxylin and eosin stained slides by a neuropathologist (L.C.K.), representative slides were chosen and immunohistochemical stains for EGFR were performed on tissue sections from the corresponding paraffin block.

### Immunohistochemistry

Four-micron thick sections were cut from formalin fixed tissue embedded in paraffin blocks and mounted onto polylysine-coated slides. Tissue sections were subjected to antigen retrieval by heating to 80-90 degrees Celsius and stained for EGFR with commercially available antisera (#M3563, clone H11, DAKO Corporation, Carpinteria, CA). Detection was performed using a standard biotin streptavidin detection system (DAKO, Carpinteria, CA). All stains were performed on the DAKO Autostainer.

### Immunohistochemical Evaluation

**Figure 1:**
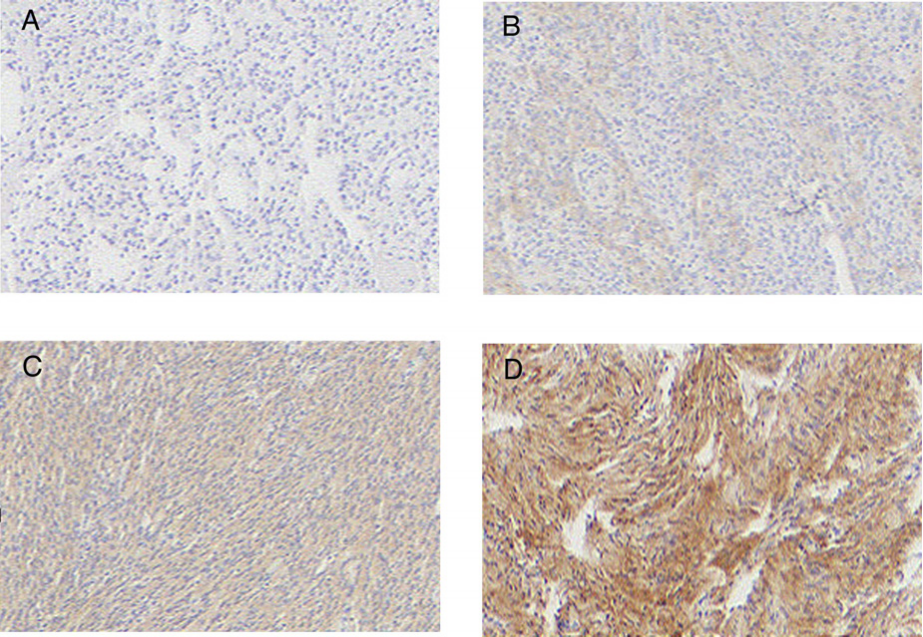
**Immunohistochemical staining intensity scores**. **A) **Meningiomas stained with anti-EGFR antisera showing negative staining. **B) **1+ staining **C) **2+ staining **D) **3+ staining. Original magnification for all images was 40 ×. Images are arranged as follows: Upper left (A), upper right (B), lower left (C), lower right (D).

Slides stained for EGFR were reviewed with the observers blinded to classification of the tumor subtype. Sections of colon cancer stained for EGFR were used as positive controls. Staining intensity was scored on a scale of 0 to 3, where 0 represents absent staining, 1-weak, 2-moderate, and 3-strong staining of the tumor specimen (Figure 1). The percentages of immunoreactive cells (staining percentage) were estimated by inspection and scored from 1 to 5, 1 (< 20% of the sample exhibiting staining); 2 (21-40% of the sample exhibiting staining); 3 (41-60% of the sample exhibiting staining); 4 (61-80% of the sample exhibiting staining), and 5 (81-100% of the specimen stained). An immunohistochemical score (IHS) was calculated as the product of an estimate of the percentage of immunoreactive cells (staining percentage (SP) score) and the estimate of the staining intensity (staining intensity (SI) score). When there was multifocal immunoreactivity and a significant difference in staining intensities between foci, a weighted average score was calculated. The raw data were converted to the IHS by multiplying the SI scores by SP scores.

### Statistical Analysis

Prior to performing the analyses, the IHS of the specimens with more than 1 slide cut from the specimen block were averaged into one score, so that each of the eighty five specimens was represented only once in the data analysis. Analyses of association of pathology according to WHO 2000 classification with SI, SP, and IHS were carried out using exact Wilcoxon tests. In the case of IHS, an exact Wilcoxon test was computed by Monte Carlo methods, using 99% confidence and 10,000 Monte Carlo simulations. All computations were completed by a statistician (T.H.) in StatXact v6.0 (Cytel Software Corporation, Cambridge, MA).

## Results

The tumors were originally classified according to the WHO 2000 classification [19], however, no revision of tumor grade is necessary when grading these tumors using WHO 2007 classification [20]. The samples were classified in accordance with pathologic grade and had the following distribution: benign 57/85 (67%), atypical 23/85 (27%), and malignant 5/85 (6%). There were a total of 24/85 (28%) of recurrent lesions: 11/57 (19%) of the benign and 13/23 (57%) of the atypical lesions. The patients were only represented once in this study; that is, the same tumor was not examined for EGFR expression upon recurrence. EGFR expression was detected in 86% of all meningioma samples tested. There was a significant association between intensity of EGFR staining and histopathologic subtype based on the exact Wilcoxon test (p = 0.002) (Table 1). The majority of samples demonstrated a moderate SI. Generally, the malignant meningiomas exhibited very low scores of intensity of EGFR staining, while benign and atypical samples demonstrated a higher intensity of staining. Specifically, 80% (4/5) of malignant samples had intensity scores of 0, and 20% (1/5) had intensity scores of 1. Conversely, only 9% (3/23) of atypical and 13% (5/57) of benign tumor samples had 0 intensity scores, whereas 23% (6/23) of atypical and 26% (13/57) of benign meningiomas had intensity scores of 1. The remaining proportions (61% (39/57) and 68% (14/23) of benign and atypical, respectively) had SI of 2 and higher (Table 2). Our data demonstrate that benign and atypical meningiomas stain more intensely than malignant meningiomas. In fact, when data from IHS for the benign and the atypical histopathologic types were combined and compared to the IHS for malignant meningiomas, we found a statistically significant association (p < 0.001) (Table 1).

**Table 1:** Comparisons of EGFR expression.

Measure	Comparison performed	p-value*
		
Staining Intensity	Benign vs. Atypical vs. Malignant	p = 0.002

	(Benign + Atypical) vs. Malignant	p < 0.001

		

Percentage Staining	Benign vs. Atypical vs. Malignant	p = 0.029
	(Benign + Atypical) vs. Malignant	p = 0.009

		

HIS	Benign vs. Atypical vs. Malignant	p = 0.004

	(Benign + Atypical) vs. Malignant	p < 0.001

* p-value based on Wilcoxon exact tests (see Methods).

**Table 2:** EGFR staining intensity of meningioma samples, n (%).

Pathology	EGFR Staining Intensity			
	***0***	***1***	***2***	***3***

Benign	5 (13)	13 (26)	38 (48)	1 (13)
Atypical	3 (9)	6 (23)	11 (66)	3 (2)
Malignant	4 (80)	1 (20)	0	0

**Figure 2:**
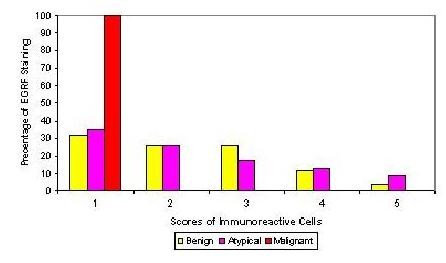
**Percentage of EGFR staining by histopathological classification**. Tumor sections were analyzed with respect to percent of each sample exhibiting staining for EGFR. The percentages of immunoreactive cells (staining percentage) were estimated by inspection and scored from 1 to 5, 1 (< 20% of the sample exhibiting staining); 2 (21-40% of the sample exhibiting staining); 3 (41-60% of the sample exhibiting staining); 4 (61-80% of the sample exhibiting staining), and 5 (81-100% of the specimen stained). The findings of the immunoreactivity for EGFR on each slide were then correlated with the meningioma histologic subtypes: benign, atypical, and malignant.

There was also a significant association between percentage of tumor cell immunoreactivity or immunoreactive cells (SP) and histopathologic subtype (p = 0.029) (Table 1). As the percentages of SP were estimated by inspection and scored from 1 to 5, 1 (< 20% of the sample exhibiting staining); 2 (21-40% of the sample exhibiting staining); 3 (41-60% of the sample exhibiting staining); 4 (61-80% of the sample exhibiting staining), and 5 (81-100% of the specimen stained), we found that while the benign and atypical meningiomas demonstrated intermediate to marked SP categories, all of the malignant meningioma samples had low scores of SP for EGFR. To illustrate this point, 100% (5/5) of the malignant samples had a 1 score of staining, while 32% (18/57) of benign and 35% (8/23) of atypical meningiomas had the same level of immunoreactivity. To examine distribution scores, please refer to Table 3 and Figure 2. When the benign and the atypical samples of meningioma were compared to the malignant specimens with respect to the distribution of immunoreactivity, a significant association was demonstrated (p = 0.009) (Table 1).

**Table 3:** Percentage of EGFR staining (immunoreactivity) of meningioma samples, n (%).

Pathology	Percentage of EGFR Staining				
	***1 (0-20%)***	***2 (21-40%)***	***3 (41-60%)***	***4 (61-80%)***	***5 (81-100%)***

Benign	18 (32)	15 (26)	15 (26)	7 (12)	2 (4)
Atypical	8 (35)	6 (26)	4 (17)	3 (13)	2 (9)
Malignant	5 (100)	0	0	0	0

The distribution of immunohistochemical scores (HIS) was also different with respect to pathologic classification, based on the exact Wilcoxon test with Monte Carlo simulation (p = 0.004) (Table 1). While this demonstrates that there was a range of IHS for the benign and the atypical histologic subtypes, the malignant tumors exhibited the lowest score and were statistically different from the benign and the atypical specimens (p < 0.001) (Table 4 and Table 1). In fact, 100% of malignant meningiomas had IHS of 0 and 1 (Table 4).

**Table 4:** Immunohistochemical Score (IHS) of meningioma samples, n (%).

Pathology	Immunohistochemical Score									
	***0***	***1***	***2***	***3***	***4***	***5***	***6***	***7***	***8***	***10***

Benign	5 (9)	4 (7)	12 (21)	7 (12)	15 (26)	0 (0)	8 (14)	0 (0)	4 (7)	2 (4)
Atypical	3 (13)	4 (17)	3 (13)	2 (9)	2 (9)	1 (4)	2 (9)	1 (4)	3 (13)	2 (9)
Malignant	4 (80)	1 (20)	0	0	0	0	0	0	0	0

## Discussion

Meningiomas are common central nervous system tumors. Although slow growing, at times, they continue to be a major cause of morbidity and mortality. Persistent risk of recurrence of these tumors is a compelling reason to seek adjuvant therapies to decrease the rates of relapse. Recent publications report an intense search for new molecular markers that may serve as potential therapeutic targets [21-28]. EGFR has emerged as one of the novel receptors expressed on the surface of a variety of cancers such as colorectal, head and neck, and lung malignancies. While its activation stimulates tumor proliferation, overexpression of EGFR in various epithelial tumors is associated with a poor patient prognosis. The notion of its function prompted development of inhibitors of EGFR which have been approved for clinical use [21-28]. Our motivation for this analysis was the fact that, to date, there is no effective pharmacologic therapy for meningioma. This study was designed to determine whether meningiomas express EGFR, and if so, to establish a correlation between the histopathologic grade of these tumors and the degree of EGFR expression.

To our knowledge, this analysis represents the largest series of meningiomas evaluated for EGFR expression in the literature to date. We demonstrated that meningiomas express EGFR and found that there was a significant association between the intensity of EGFR staining and tumor grade. While the majority of samples had a moderate level of staining intensity, the malignant tumor grade exhibited the lowest scores. Our data demonstrate significantly greater degree of EGFR expression in benign and atypical meningiomas as compared to the malignant meningiomas. With respect to percentage of immunorectivity, the malignant grade of meningioma revealed lower scores in contrast with the benign and atypical samples. Further, malignant tumors exhibited the lowest immunohistochemical scores and were different from the scores of the benign and the atypical specimens in a statistically significant fashion. Therefore, we conclude that EGFR expression is inversely correlated with tumor grade in meningiomas.

Some investigators, utilizing ligand-binding techniques, demonstrated a broad range of EGFR expression in meningiomas, varying from approximately 30% to 100% [29-32]. The discrepancies in the literature regarding the expression levels of EGFR in meningiomas may be accounted for by the different techniques used in each of these studies. In our study, we determined the expression of EGFR in meningiomas by immunohistochemical analysis of archival tissue, and EGFR expression was detected in 86% of all meningiomas tested. While the majority of studies previously reported a specific EGFR immunoreactivity in the vascular endothelial cells of meningiomas [33-36], there were others that demonstrated no such correlation [37-40]. We presented our data with respect to the percent of immunoreactivity (SP) in our meningioma samples and found a significant association between percentage of immunoreactive cells staining for EGFR and histopathologic subtype.

What does overexpression of EGFR in tumors indicate? Carroll et al provide an explanation by examining EGFR expression in human meningiomas by Western blot and immunohistochemical analyses [33]. The authors speculate that activation of EGFR is not a result of any mutations of the EGFR, but it is secondary to autocrine/paracrine stimulation by their endogenous ligands, EGF and TGF alpha, which are also expressed in meningiomas and may contribute to meningothelial cell proliferation [33]. Kong et al also supports that EGFR receptors are regulated by autocrine mechanism [41]. An alternative theory is that the benign histologic subtypes have a more efficient autocrine/paracrine stimulation, which makes them significantly different from all other types not only in their behavior but also in their expression of EGFR [33]. Lusis et al in a report evaluating the expression of EGFR in 41 meningiomas identified a relatively higher incidence of EGFR expression among incidental asymptomatic meningiomas discovered at autopsy compared with those removed during surgical treatment [42]. This finding is consistent with the EGFR pathway of meningioma growth stimulation resulting in a relatively less aggressive tumor [42]. Smith et al reported that absence of EGFR expression correlates with poor prognosis in patients with meningioma [43]. Although it may be expected that increased expression of EGFR would provide a growth advantage and thus correlate with a worse prognosis, the opposite was true in his series. This finding does not necessarily cast doubt on the theory that EGFR is involved in the development of meningiomas, as considerable evidence implicates this receptor in tumor development. Instead, these data suggest that in tumors lacking EGFR expression, other even more potent growth-stimulatory mechanisms may exist [43].

EGFR activation increases resistance to apoptosis, promotes angiogenesis, and impairs immune surveillance; hence, intervention with an EGFR inhibitor may decrease tumorigenic progression in patients with this disease. While radiation therapy plays an important role in the management of meningioma, an association between high EGFR expression and clinical radioresistance has been reported in patients with cancer. Correlation between EGFR overexpression and response to radiotherapy has been well described in human head and neck cancers [32]. Furthermore, overexpression of EGFR may act as an independent prognostic factor for relapse and recurrence of disease. Ang et al reported on patients with squamous cell carcinomas of the head and neck (SCCHN) as part of the correlative biomarker study, where the overall survival (OS) and disease-free survival (DFS) rates of patients with high EGFR-expressing SCCHN were highly significantly lower and the local recurrence (LR) relapse rate was significantly higher compared with those of patients with low EGFR-expressing SCCHN [44]. Multivariate analysis showed that EGFR expression was an independent determinant of OS and a robust independent predictor of LR relapse. The data suggest that EGFR immunohistochemistry should be considered for selecting patients for more aggressive combined therapies or enrollment into trials targeting EGFR signaling pathways [44]. A phase III randomized clinical trial evaluated the addition of cetuximab (Erbitux TM) to high dose radiation in patients with locoregionally advanced SCCHN and demonstrated a statistically significant prolongation of OS in the combined modality arm versus radiation alone [45,46]. EGFR overexpression was also found to be a significant and independent prognostic indicator for OS after radiation therapy in patients with astrocytic gliomas [47]. The addition of EGFR inhibitors to patients receiving radiation therapy has not been found to significantly increase the toxicity of treatment [32,45,46]. Most toxicities associated with cetuximab in the treatment of head and neck cancers are low grade and cutaneous. The rationale for combination of inhibitors of EGFR with ionizing radiation is, therefore, a potentially attractive combination for recurrent or benign meningioma.

There are now several EGFR inhibitors- Herceptin (Trastuzumab), Erbitux (IMC-C225, cetuximab), Tarceva (OSI-774, erlotinib), Iressa (ZD 1839), Maztuzumab (EMD 72000) - which exhibit anti-cancer activity and are being used in clinical practice for the tumors of breast, colon, head and neck, lung, and others. Although the precise mechanism by which EGFR inhibitors exert their anti-cancer effect remains unknown, compelling evidence exists to further explore whether inhibitors of EGFR will be of clinical benefit to patients with benign/low-grade or recurrent meningioma, which represent the vast majority of patients. The association of EGFR and meningioma grade is a potential new avenue for therapeutic intervention with selective EGFR inhibitors, either as an adjuvant treatment or in combination with radiation therapy. Additional clinical studies will be needed before inhibitors of EGFR can be incorporated into clinical practice.

## Competing interests

The authors declare that they have no competing interests.

## Authors' contributions

AGW carried out the conception, design and coordination of the study, scoring slides, analysis and interpretation of the data and drafting of the manuscript. APD participated in the conception and design of the study. MW participated in identifying cases, acquisition of the data and in drafting the manuscript. JI participated in the acquisition of the data and in drafting the manuscript. TH participated in the design of the study and performed the statistical analysis. EHH and AP participated in case selection and provided patient material for analysis.  DAW participated in case selection and identification.  LK carried out conception, coordination, pathologic interpretation, grading, scoring of immunohistochemical stains, and drafting of the manuscript. All authors read and approved the final manuscript.

## References

[B1] Rachlin JR Rosenblum ML Etiology and biology of meningiomas 1991 New York: Raven Press

[B2] DeMonte F Current management of meningiomas Oncology 2001 9 83 96 7718443

[B3] Jaaskelainen J Haltia M Servo A Atypical and anaplastic meningiomas: radiology, surgery, radiotherapy, and outcome Surg Neurol 1986 25 3 233 242 10.1016/0090-3019(86)90233-8 3945904

[B4] Mirimanoff RO Dosoretz DE Linggood RM Ojemann RG Martuza RL Meningioma: analysis of recurrence and progression following neurosurgical resection J Neurosurg 1985 62 1 18 24 10.3171/jns.1985.62.1.0018 3964853

[B5] Jaaskelainen J Haltia M Servo A Atypical and anaplastic meningiomas: radiology, surgery, radiotherapy, and outcome Surg Neurol 1986 25 3 233 242 10.1016/0090-3019(86)90233-8 3945904

[B6] Barbaro NM Gutin PH Wilson CB Sheline GE Boldrey EB Wara WM Radiation therapy in the treatment of partially resected meningiomas Neurosurgery 1987 20 4 525 528 10.1227/00006123-198704000-00003 3587542

[B7] Goldsmith BJ Wara WM Wilson CB Larson DA Postoperative irradiation for subtotally resected meningiomas. A retrospective analysis of 140 patients treated from 1967 to 1990 J Neurosurg 1994 80 2 195 201 10.3171/jns.1994.80.2.0195 8283256

[B8] Taylor BW Jr Marcus RB Jr Friedman WA Ballinger WE Jr Million RR The meningioma controversy: postoperative radiation therapy Int J Radiat Oncol Biol Phys 1988 15 2 299 304 3403313 10.1016/s0360-3016(98)90008-6

[B9] Ennis BW Lippman ME Dickson RB The EGF receptor system as a target for antitumor therapy Cancer Invest 1991 9 553 562 10.3109/07357909109018953 1933488

[B10] Bucci B D'Agnano I Botti C Mottolese M Carico E Zupi G Vecchione A EGF-R expression in ductal breast cancer: Proliferation and prognostic implications Anticancer Res 1997 17 769 774 9066618

[B11] Salomon DS Brandt R Ciardiello F Normanno N Epidermal growth factor-related peptides and their receptors in human malignancies Crit Rev Oncol Hematol 1995 19 183 232 10.1016/1040-8428(94)00144-I 7612182

[B12] Ruben Grandis J Melhem MF Barnes EL Tweardy DJ Quantitative immunohistochemical analysis of transfroming growth factor alpha and epidermal growth factor receptor in patients with squamous cell carcinoma of the head and neck Cancer 1996 78 1284 1292 10.1002/(SICI)1097-0142(19960915)78:6<1284::AID-CNCR17>3.0.CO;2-X 8826952

[B13] Rieske P Kordek R Bartkowiak J Debiec-Rychter M Biernat W Liberski PP A comparative study of epidermal growth factor receptor (EGFR) and MDM2 gene amplification and protein immunoreactivity in human glioblastomas Pol J Pathol 1998 49 145 149 9810172

[B14] Goike HM Asplund AC Pettersson EH Liu L Sanoudou D Collins VP Acquired rearrangement of an amplified epidermal growth factor receptor (EGFR) gene in human glioblastoma xenograft J Neuropathol Exp Neurol 1999 58 697 701 10.1097/00005072-199907000-00003 10411339

[B15] Messa C Russo F Caruso MG Di Leo A EGFR, TGF-alpha, and EGF-R in human colorectal adenocarcinoma Acta Oncol 1998 37 285 289 10.1080/028418698429595 9677101

[B16] Libermann TA Razon N Bartal AD Yarden Y Schlessinger J Soreq H Expression of epidermal growth factor receptors in human brain tumors Cancer Res 1984 44 753 760 6318976

[B17] Westermark B Magnusson A Heldin CH Effects of epidermal growth factor on membrane motility and cell locomotion in cultures of human clonal glioma cells J Neurosci Res 1982 8 491 507 10.1002/jnr.490080236 6296418

[B18] Weisman AS Villemure JG Kelly PA Characterization of epidermal growth factor receptor in human meningioma Cancer Res 1987 47 2172 2176 3493842

[B19] Kleihues P Cavenee WK Pathology and genetics, tumours of the nervous system International Agency for Research on Cancer. Lyon, France 2000 176 184

[B20] Louis DN Ohgaki H Weistler OD Cavenee WK Burger PC Jouvet A Scheithauer BW Kleihues P WHO Classification of Tumours of the Central Nervous System 2007 3 Geneva: WHO Press 10.1007/s00401-007-0243-4 PMC1929165 17618441

[B21] Andersson U Guo D Malmer B Bergenheim AT Brännström T Hedman H Henriksson R Epidermal growth factor receptor family (EGFR, ErbB2-4) in gliomas and meningiomas Acta Neuropathol 2004 108 2 135 142 10.1007/s00401-004-0875-6 15148612

[B22] Bianco R Damiano V Gelardi T Ciardiello F Tortora G Rational combination of targeted therapies as a strategy to overcome the mechanisms of resistance to inhibitors of EGFR signaling Curr Pharm 2007 13 33 3358 3367 10.2174/138161207782360537 18045190

[B23] Lee J Chu E First-line use of anti-epidermal growth factor receptor monoclonal antibodies in metastatic colorectal cancer Clin Colorectal Cancer 2007 6 Suppl 2 S42 46 10.3816/CCC.2007.s.001 18021486

[B24] Astsaturov I Cohen RB Harari P EGFR-targeting monoclonal antibodies in head and neck cancer Curr Cancer Drug Targets 2007 7 7 650 665 10.2174/156800907782418365 18045070

[B25] Blick SK Scott LJ Cetuximab: a review of its use in squamous cell carcinoma of the head and neck and metastatic colorectal cancer Drugs 2007 67 17 2585 2607 10.2165/00003495-200767170-00008 18034592

[B26] Gridelli C Rossi A Maione P Colantuoni G Del Gaizo F Ferrara C Nicolella D Guerriero C Erlotinib in no-small-cell lung cancer Expert Opin Pharmacother 2007 8 15 2579 2592 10.1517/14656566.8.15.2579 17931092

[B27] Thatcjer N The place of targeted therapy in the patient management of non-small cell lung cancer Lung Cancer 2007 57 Suppl 2 S18 23 10.1016/S0169-5002(07)70423-3 17686441

[B28] Piccart-gebhart MJ Procter M Leyland-Jones B Goldhirsch A Untch M Smith I Gianni L Baselga J Bell R Jackisch C Cameron D Dowsett M Barrios CH Steger G Huang CS Andersson M Inbar M Lichinitser M Láng I Nitz U Iwata H Thomssen C Lohrisch C Suter TM Rüschoff J Suto T Greatorex V Ward C Straehle C McFadden E Dolci MS Gelber RD Trastuzumab after adjuvant chemotherapy in Her2-positive breast cancer N Engl J Med 2005 353 16 1659 1672 10.1056/NEJMoa052306 16236737

[B29] Sanfilippo JS Rao CV Guarnascelli JJ Detection of epidermal growth factor and transforming growth factor alpha protein in meningiomas and other tumors of central nervous system in human beings Surg Gynecol Obstet 1993 177 488 496 8211601

[B30] Westphal M Herrmann HD Epidermal growth factor receptors on cultured human meningioma cells Acta Neurochir 1986 83 62 66 10.1007/BF01420510 3492089

[B31] Weisman AS Villemure JG Kelly PA Regulation of DNA synthesis and growth of cells derived from primary human meningiomas Cancer Res 1986 46 2545 2550 3697993

[B32] Maurizi M Almadori G Ferrandina G Prognostic significance of epidermal growth factor receptor in laryngeal squamous cell carcinoma Br J Cancer 1996 74 1253 1257 8883413 10.1038/bjc.1996.525 PMC2075924

[B33] Carroll RS Black PM Zhang J Kirsch M Percec I Lau N Guha A Expression and activation of epidermal growth factor receptors in meningiomas J Neurosurg 1997 87 2 315 323 10.3171/jns.1997.87.2.0315 9254099

[B34] Diedrich U Lucius J Baron E Behnke J Pabst B Zoll B Distribution of epidermal growth factor receptor gene amplification in brain tumours and correlation to prognosis J Neurol 1995 242 10 683 688 10.1007/BF00866920 8568531

[B35] DiCarlo A Mariano A Macchia PE Moroni MC Beguinot L Macchia V Epidermal growth factor receptor in human brain tumors J Endocrinol Invest 1992 15 1 31 37 1560188 10.1007/BF03348650

[B36] Johnson MD Horiba M Winnier AR Artiaga CL The epidermal growth factor receptor is associated with phospholipase C-gamma 1 in meningiomas Hum Pathol 1994 25 2 146 153 10.1016/0046-8177(94)90270-4 8119714

[B37] Jones NR Rossi ML Gregoriou M Hughes JT Epidermal Growth Factor Receptor expression in 72 meningiomas Cancer 1990 66 1 152 155 10.1002/1097-0142(19900701)66:1<152::AID-CNCR2820660127>3.0.CO;2-5 2354402

[B38] Dorward NL Hawkins RA Whittle IR Epidermal growth factor receptor activity and clinical outcome in glioblastoma and meningioma Br J Neurosurg 1993 7 2 197 199 10.3109/02688699309103479 8388222

[B39] Shiurba RA Eng LF Vogel H Lee YL Horoupian DS Urich H Epidermal growth factor receptor in meningiomas is expressed predominantly on endothelial cell Cancer 1988 62 10 2139 2144 10.1002/1097-0142(19881115)62:10<2139::AID-CNCR2820621013>3.0.CO;2-G 3052782

[B40] Camby I Nagy N Rombaut K Gras T Duponchelle C Pasteels JL Brotchi J Kiss R Salmon I Influence of epidermal growth factor and gastrin on the cell proliferation of human meningiomas versus astrocytic tumors maintained as ex vivo tissue cultures Neuropeptides 1997 31 3 217 225 10.1016/S0143-4179(97)90051-2 9243517

[B41] Kong YG Su CB Ren ZY Wang RZ Measurement of epidermal growth factor receptor concentration in the pre-and post-operative serum in patients with meningiomas Zhongguo Yi Xue Ke Yuan Xue Bao 2002 24 4 427 429 12905669

[B42] Lusis EA Chicoine MR Perry A A high throughput screening of meningioma biomarkers using a tissue microarray J Neurooncol 2005 73 219 223 10.1007/s11060-004-5233-y 15980972

[B43] Smith JS Lal A Harmon-Smith M Bollen AW McDermott MW Association between absence of epidermal growth factor receptor immunoreactivity and poor prognosis in patients with atypical meningioma J Neurosurg 2007 106 1034 1040 10.3171/jns.2007.106.6.1034 17564176

[B44] Ang KK Berkley BA Tu X Impact of Epidermal Growth Factor Receptor expression on survival and pattern of relapse in patients with advanced head and neck carcinoma Cancer Res 2002 62 7350 7356 12499279

[B45] Bonner JA Giralt J Harari PM Cetuximab prolongs survival in patients with locoregionally advanced head and neck cancer: A phase III study of high dose radiation therapy with and without cetuximab J Clin Oncology, 2004 ASCO Annual Meeting Proceedings (Post-Meeting Edition) 22 14S 5507

[B46] Bonner JA Harari PM Giralt J Azarnia N Shin DM Cohen RB Jones CU Sur R Raben D Jassem J Ove R Kies MS Baselga J Youssoufian H Amellal N Rowinsky EK Ang KK Radiotherapy plus cetuximab for squamous-cell carcinoma of the head and neck N Engl J Med 2006 9; 354 6 567 78 10.1056/NEJMoa053422 16467544

[B47] Zhu A Shaeffer J Leslie S Epidermal growth factor receptor: An independent predictor of survival in astrocytic tumors given definitive irradiation Int J Radiation Oncology Biol Phys 1996 34 809 815 10.1016/0360-3016(95)02184-1 8598357

